# Lack of change in CA1 dendritic spine density or clustering in rats following training on a radial-arm maze task

**DOI:** 10.12688/wellcomeopenres.15745.2

**Published:** 2020-05-15

**Authors:** Emma Craig, Christopher M. Dillingham, Michal M. Milczarek, Heather M. Phillips, Moira Davies, James C. Perry, Seralynne D. Vann

**Affiliations:** 1 School of Psychology, Cardiff University, Cardiff, CF10 3AT, UK

**Keywords:** Golgi stain, hippocampus, spatial memory, spinogenesis

## Abstract

**Background:** Neuronal plasticity is thought to underlie learning and memory formation. The density of dendritic spines in the CA1 region of the hippocampus has been repeatedly linked to mnemonic processes. Both the number and spatial location of the spines, in terms of proximity to nearest neighbour, have been implicated in memory formation. To examine how spatial training impacts synaptic structure in the hippocampus, Lister-Hooded rats were trained on a hippocampal-dependent spatial task in the radial-arm maze.

**Methods: **One group of rats were trained on a hippocampal-dependent spatial task in the radial arm maze. Two further control groups were included: a yoked group which received the same sensorimotor stimulation in the radial-maze but without a memory load, and home-cage controls. At the end of behavioural training, the brains underwent Golgi staining. Spines on CA1 pyramidal neuron dendrites were imaged and quantitatively assessed to provide measures of density and distance from nearest neighbour.

**Results:** There was no difference across behavioural groups either in terms of spine density or in the clustering of dendritic spines.

**Conclusions:** Spatial learning is not always accompanied by changes in either the density or clustering of dendritic spines on the basal arbour of CA1 pyramidal neurons when assessed using Golgi imaging.

## Introduction

The hippocampus plays a vital role in spatial learning and memory ^1, 2^. Since the discovery of place cells in the Cornu Ammonis 1 (CA1) field of the hippocampus ^3^, this subregion has become a major focus of research into spatial memory. Numerous studies, across species, have identified a role for CA1 in spatial learning and memory. For example, rats with CA1 lesions are impaired on spatial memory tasks ^4^. Furthermore, the extent of pyramidal neuron loss within CA1 has been found to correlate with performance on a T-maze task, regardless of overall hippocampal damage ^5^. This is consistent with findings from patient studies where extent of damage to CA1 correlates with impairment on a virtual place learning task ^6^.

Learning and memory is supported by neural plasticity, whereby learning episodes elicit subcellular morphological changes, facilitating the long-term representation of the event. Neural plasticity includes the experience-dependent modification of dendritic spines. Excitatory neuronal firing can increase numbers of CA1 dendritic spines both *in vivo* and *in vitro* ^7, 8^. A number of behavioural tasks have been found to bring about an increase in CA1 spine density, with changes most pronounced on the basal arbors of CA1 pyramidal neurons ^9^. While there is a long-standing association between memory and spine density, more recent studies have also highlighted the importance not only of the overall number but also the location of the spines. Neighbouring synapses will result in greater depolarisation of the neuron when simultaneously activated, thus providing a mechanism for greater processing capacity ^10^. Consistent with this idea, learning has been shown to result in dendritic spines that are located in close proximity, i.e. clustered.

To date, most studies have focused on spine clustering in cortex ^11^, although there is evidence that watermaze training also affects spine clustering in CA1 ^3^. However, tasks carried out in the watermaze are typically aversive, so changes can be difficult to interpret as stress has also been shown to affect spine density ^12– 14^. Furthermore, it is difficult to identify suitable behavioural controls for watermaze tasks ^15^. We therefore assessed spine density and clustering in rats that had been trained on appetitive tasks, i.e. working memory version of the radial-arm maze task. This task, and species, was chosen as CA1 activity has been associated with performance on this task ^16– 18^ and previous studies in rats have reported increased spine density following radial-arm maze training ^19– 21^. Mahmmoud *et al.* ^21^ also found a significant correlation between spine density of CA1 basal arbors and errors on the radial-arm maze task. These results not only suggest that spine density increases following training on the radial-arm maze but that it is also directly linked to performance. Following standard training on the radial-arm maze task, animals underwent further testing where the maze was rotated mid-trial, to ensure animals were performing the task using extramaze cues. Two control groups were included, one behavioural control group that was trained to run up and down one arm of the maze. Behavioural control and experimental animals were, therefore, matched for sensorimotor stimulation and rewards received but differed in terms of mnemonic demand. A further home-cage control group was included, which comprised animals that were age-matched but had undergone no behavioural training.

The current study tested the hypothesis that spatial memory training results in increased spine density and clustering in the CA1 subregion of the hippocampus. As such, we would expect to see differences between the spatial memory group and both of the control groups.

## Methods

### Animals

Thirty naïve adult male Lister-Hooded rats (Harlan, UK) were involved in the study. The rats were approximately 3 months of age at the start of the experiment and maintained around 300g for the extent of the experiment (approximately 5 weeks). Rats were housed in pairs under diurnal light conditions (14 h light/10 h dark) and any testing was carried out at a regular time during the light phase. Cages were plastic-based with metal bars forming the lid. Sawdust covered the floor of the cage and a cardboard tube was placed within each cage. During the behavioural testing period, animals were food deprived but their body weight did not fall below 85% of free feeding weight. Animals were given access to water throughout. Animals were habituated to handling before commencing the study. The experiment was carried out in accordance with UK Animals (Scientific Procedures) Act, 1986 and associated guidelines. All efforts were made to ameliorate any suffering of animals. We used an appetitive behavioural task, rather than an aversive task, to minimise stress.

***Sample size.*** Thirty animals were used in total, ten in each experimental condition. This number was arrived at on the basis of previously published studies using similar approaches and addressing similar questions ^19– 22^.

***Animal allocation.*** There were three experimental groups: a home-cage group that was food restricted but did not undergo behavioural training; a spatial memory group that was trained on a working memory version of the radial-arm maze task; finally, a yoked-control group were matched for the sensorimotor aspects of radial-arm maze testing but without the memory load by simply running up and down one arm of the maze for food rewards. The animals were allocated randomly to the experimental groups at the outset of the experiment on the basis of rat number so for every three rats there would be one animal in each experimental condition. The only constraining factor was that the home-cage controls were housed together while the other cages contained one yoked control and one spatial memory rat. The spatial memory and yoked control animals were interleaved for behavioural testing. Once the tissue had been processed all slides were anonymised such that all data collection and analysis was carried out with the experimenter blind to experimental group.

### Radial-arm maze task

***Apparatus.*** The radial-arm maze consisted of an octagonal central platform with eight equally spaced arms radiating from the central platform. Food wells were located at the end of each arm. The floors of the maze were made of wood and painted white while the walls were made of transparent Perspex. Each arm had a Perspex sliding door, attached to a pulley system, enabling the experimenter to control access to and from the central platform. The entire maze was placed on wheels so that it could be easily rotated. Geometric shapes and other high contrast stimuli were located on the walls.

***Behavioural procedure.*** Rats in the spatial memory group and the yoked controls were brought from the holding room to the testing room in pairs in an opaque carrier case. Rats underwent four habituation sessions where they could freely explore the maze for 10 minutes, for the first two days with all the doors raised and for the second two days with the doors opened and closed. For the first habituation session, rats were placed in the maze in pairs; for the remaining three habituation sessions they were habituated individually. For all habituation sessions, sucrose reward pellets (45mg, LabDiet, St Louis, Missouri, US) were scattered down the arms.

In the training phase for the spatial memory group, all eight arms were baited with a single reward pellet. The rat was placed on the central platform, with all doors closed. The experimenter then opened all the doors allowing the animal to choose an arm to enter. After eating the reward pellet the animal returned to the central platform and the doors were closed for 10 seconds before being opened again, allowing the rat to make another choice. This continued until all arms were visited or until a 10-minute time limit was reached. The optimal strategy involves retrieving all reward pellets from all 8 arms without entering previously entered arms. An error was scored if a rat entered any arm more than once. Once animals had learnt the standard task, after 12 sessions, a rotation stage was included to ensure animals were using spatial cues to perform the task. The first part of the testing session was identical to the standard version of the task. However, after four correct choices were made, the animal was removed from the maze and the maze was rotated 45 degrees. This was either clockwise or anti clockwise on alternate days. The remaining food pellets were moved so that they were in the same position in relation to the extra-maze cues. Following this, the doors of the maze were re-opened and the animal was allowed to complete the trial, i.e. retrieve the remaining four rewards. After this there was a test phase in which the rat was returned to the central platform until the remaining four reward pellets were retrieved. The animals received six rotation sessions.

Yoked animals spent the same overall amount of time in the radial-arm maze as their counterparts, and received the same number of rewards, but they only had access to one arm of the maze, which remained the same throughout training.

### Golgi staining

Ninety minutes after the behavioural animals completed the final test session, they were anaesthetised with sodium pentobarbital and transcardially perfused with 0.1 M phosphate buffer saline (PBS) followed by 4% paraformaldehyde in 0.1 M PBS (PFA).

Golgi staining was carried out using the FG Rapid GolgiStain Kit based on the Golgi-Cox impregnation technique. For this, the brains were rinsed in distilled water before being immersed whole in a solution containing mercuric chloride, potassium dichromate and potassium chromate (kit solutions A+B) and stored in darkness at room temperature for approximately 2 weeks with gentle agitation. Following this, the brains were transferred into kit solution C for 1 week at 40 °C and then sliced in the coronal plane with a cryostat (thickness 150 *µ*m). The slices were mounted onto subbed microscope slides and stored in darkness for 48 hours. The sections were then rinsed in distilled water twice, for 2 minutes each, and placed in a mixture of kit solution D, E and distilled water (proportioned 1:1:2) for 8 minutes. Finally, sections were cleared in xylene for 4 minutes and coverslipped using DPX mounting medium.

### Image analysis

Image stacks from Golgi stained slices were obtained using a DM 6000 Leica microscope with a 100x oil-immersion objective (NA 1.4; Leica, Germany) attached to a Leica digital camera (Leica, DFC350 FX). Image stacks were collected with a 0.2µm step size resulting in approximately 30 – 90 images per stack. Microscope and camera settings were adjusted using the Leica Application Suite image acquisition software.

**Figure 1.**
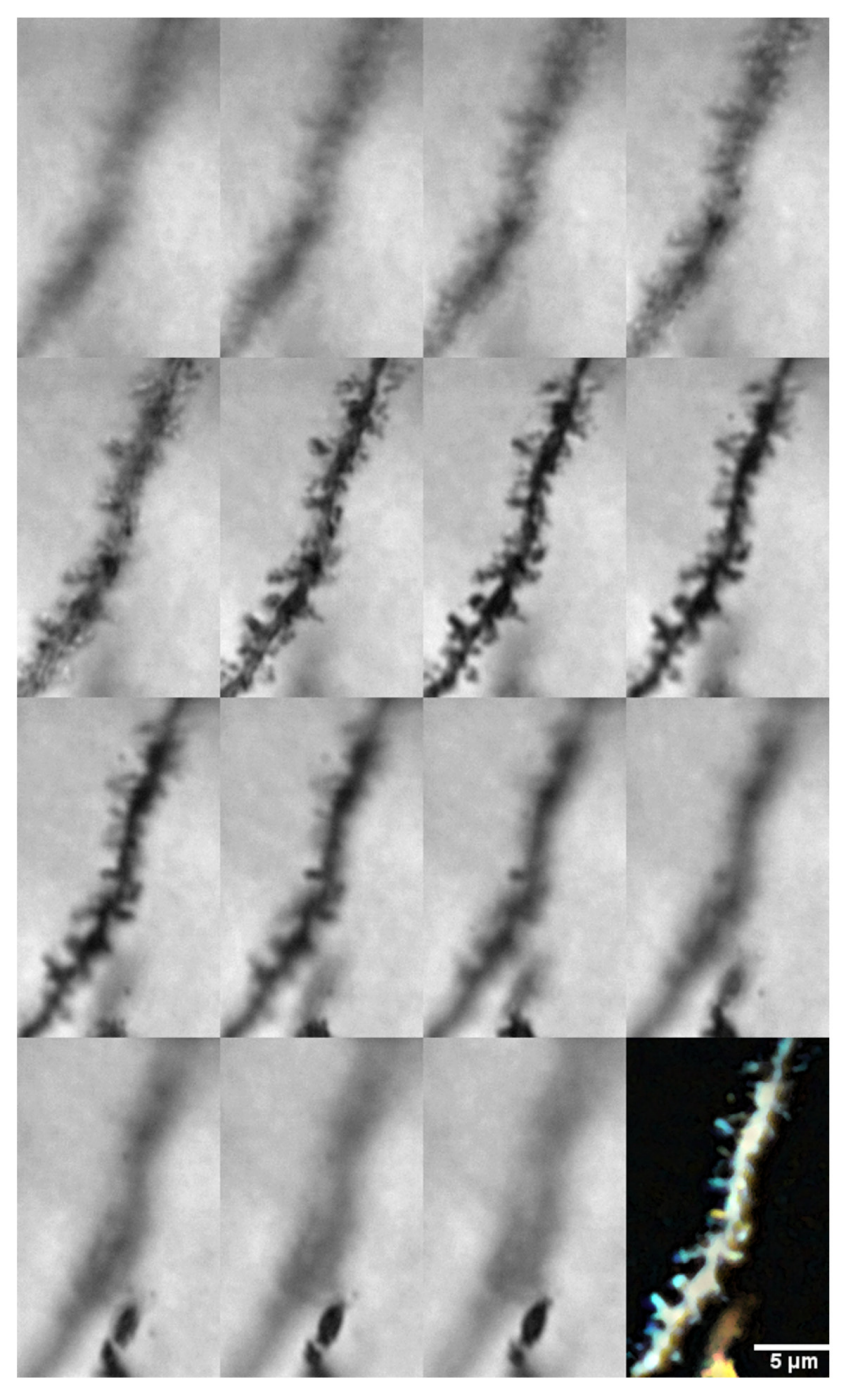
Representative example of an image stack from Golgi-stained dendritic segment of a CA1 basal arbor before and after image processing used for subsequent spine density and clustering analysis.

Approximately 20 dendritic images were collected for each brain. The dorsal CA1 region of the hippocampus was targeted, extending from 2.7mm to 4.6mm posterior to bregma ^23^. Suitable basal dendritic arbors were selected according to the eligibility criteria of previous studies ^19, 22^. Segments must be intact and clearly visible (i.e. unobscured by staining artefacts) and isolated from other stained neurons; segments must be fully impregnated by the Golgi stain; segments could not belong to the primary dendritic branch but must be selected from secondary or higher order branches; segments starting and ending extremities were at least 10 *µ*m away from a dendritic branching point or end; the beginning of a segment had to start from a point equidistant between two spines; only one segment per neuron was counted ( Figure 1).

ImageJ software (Fiji version 1.51, https://imagej.net/) and the Simple Neurite Tracer plugin were used to invert greyscale images and to measure out and crop 20–25 *μ*m dendrite section z-stack images. The images were re-inverted for subsequent processing. Cropped z-stacks were then filtered and sharpened using a custom macro to be finally flattened into a single layer using the in-built temporal colour code hyperstack projection method. The subsequent processed image represented spines at different depths using different colours. This ensured that spines that may otherwise be hidden were not missed. However, due to the opaque nature of the Golgi stain, spines that are above or below the dendrite with respect to the field of view cannot be visualised. Consistent with previous reports, no attempt was made to count or correct for these inaccessible spines during the counting process (Harland *et al.* ^19^). Spines were counted manually, and the Cartesian coordinates of identified spines were transformed onto a 1D map of the dendritic branch. During counting, spines with two or more heads (i.e. branched spines) were counted as one spine. Spine density (number of spines / 10 *μ*m dendrite length) and mean nearest neighbour (distance to the nearest spine) for each segment were calculated within ImageJ and imported into RStudio to derive mean case values. In total, 537 CA1 segments were included, from which 6504 spines were counted ^24^.

### Statistical analyses

Statistical analyses were carried out using SPSS (version 25, IBM corporation). The threshold for significance was set at *p* < 0.05 unless otherwise specified, i.e. corrected for multiple comparisons. In addition to classical hypothesis testing, the default Bayes factor was calculated to quantify the relative evidence for the null hypothesis (H0) compared to the alternative hypothesis (H1). The Bayes Factor (BF10), provides a continuous measure of evidence where a BF10 of 1 indicates that the findings are equally likely under H0 and H1, a BF10 less than 1 indicates support for H0 over H1, and BF10 greater than 1 indicates support for H1 over H0 ^25^. For example, A BF10 of greater than or equal to 3 suggests that the data are 3x more likely under H1 than H0 and could be considered ‘substantial’ evidence for H1. In comparison, a BF10 of 0.1 suggests the data are 10x more likely under H0 than H1 and could be considered ‘substantial’ evidence for H0 ^26^. A BF10 range between 1/3 – 3 could be interpreted as ‘anecdotal’ evidence for the H0 or H1 ^26^. Here it is important to note that verbal labels used to categorise different Bayes factors can be useful to facilitate scientific communication, but caution is needed due to the arbitrary nature of these labels and the continuous nature of the Bayes factor ^26^. Default Bayes Factors were calculated using JASP (version 0.11.1).

## Results

Due to incomplete staining, only 24 out of 30 cases were suitable for spine density analysis (n=8 in each group); only these animals were included in subsequent analyses.

### Behaviour

**Figure 2.**
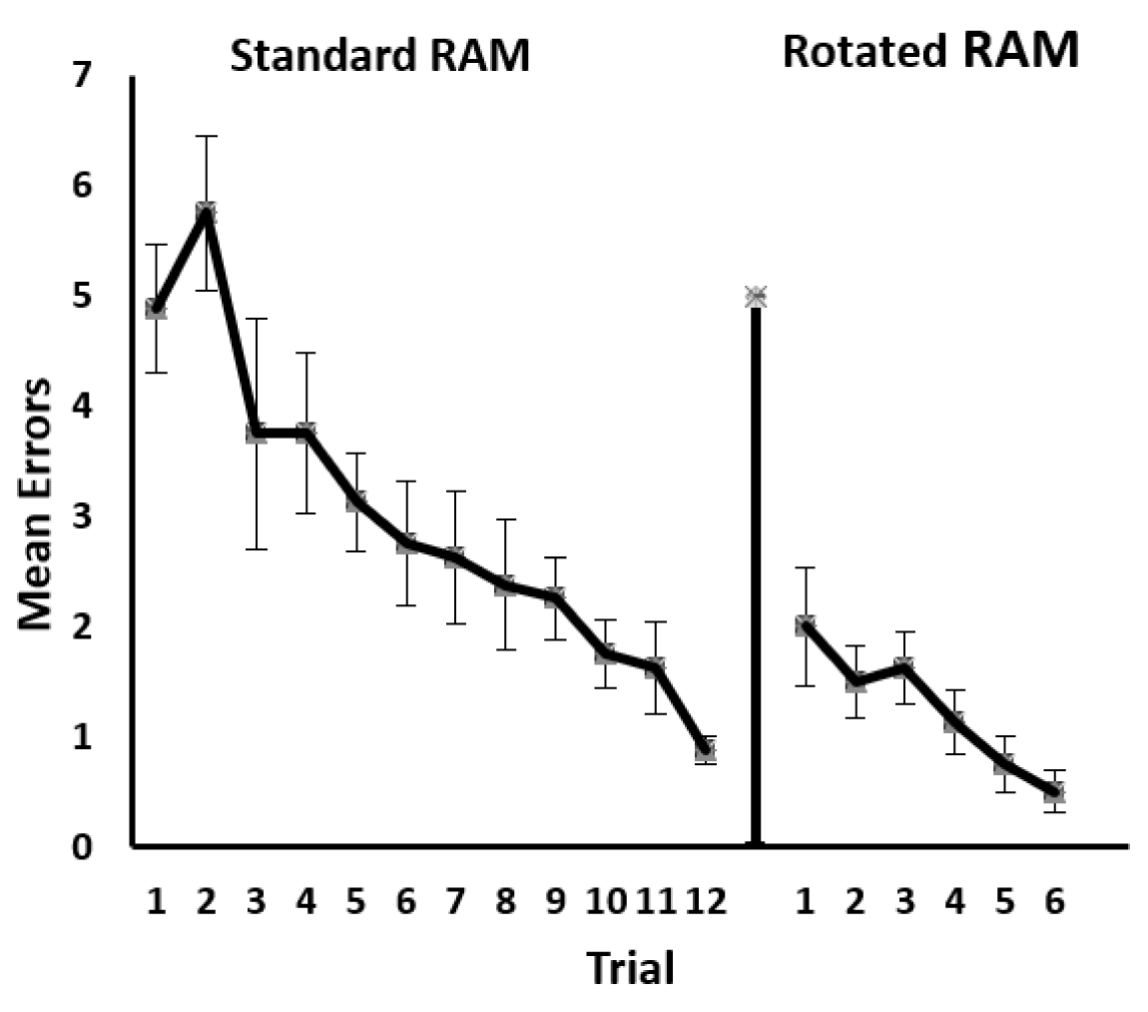
Radial-arm maze (RAM) training. There was a significant reduction in mean number of errors in both the standard and rotated phases of the task. Error bars are +/- the SEM.

A repeated one-way ANOVA showed a significant improvement in performance across training in the radial-arm maze task, both on the standard (F(11) = 6.21, *p* < 0.01) and the rotated variants (F(5) = 3.54, *p* = 0.04) ( Figure 2) ^24^.

### Spine density and clustering

**Figure 3.**
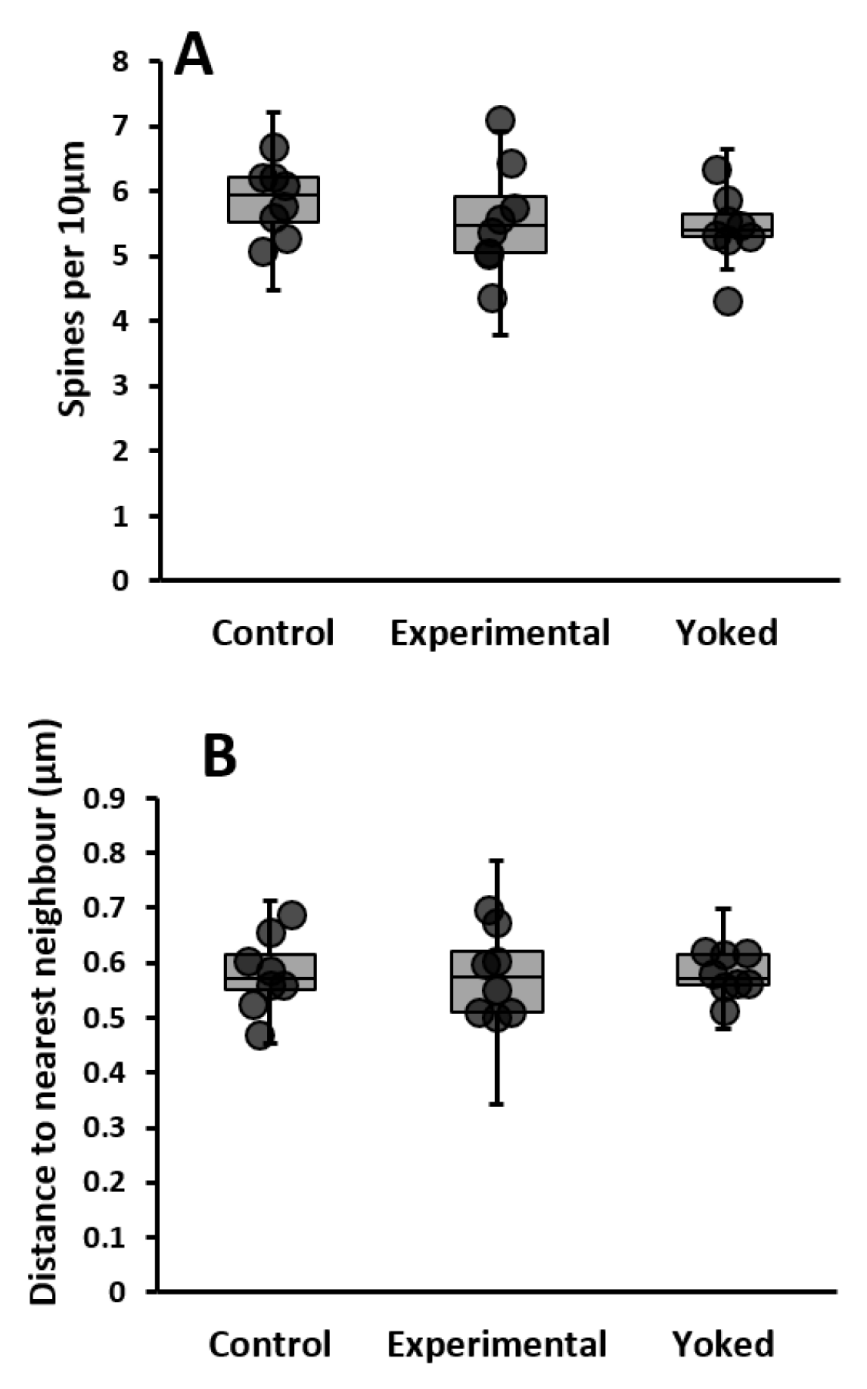
Spine morphology of CA1 basal dendrites for the home-cage control (Control), spatial memory (Experimental) and yoked-control (Yoked) groups. ( **A**) Spine density per 10 *µ* of basal dendrites in the CA1 region was not significantly different between groups. ( **B**) Mean distance between spines and their nearest neighbour (i.e., clustering) was not significantly different between groups. The central line in each box indicates the median value. The box extends from the first to the third quartile range. The whiskers extend 1.5x the interquartile range. Individual data points are shifted along the x-axis to aid visualisation of overlapping data points.

There were 191 sections with 2401 spines in the home-cage control group (n=8), 184 sections with 2119 spines in the yoked-control group (n=8) and 162 sections with 1984 spines in the spatial memory group (n=8) ^24^. One-way between-group ANOVAs found no significant difference between groups for spine density (F(2) = 1.65, *p* > 0.1, BF10 = 0.447) or mean distance to nearest neighbour, i.e., clustering (F(2) = 0.49, *p* > 0.5, BF10 = 0.245; Figure 3). To rule out the possibility that mean nearest neighbour simply reflected spine density, we investigated whether these values co-varied using Pearson’s correlation. No relationship was found ( *r* = -0.064, *p* = 0.79), indicating that spines were distributed non-randomly. Accounting for spine density by dividing mean nearest neighbour by spine density did not affect the results.

**Figure 4.**
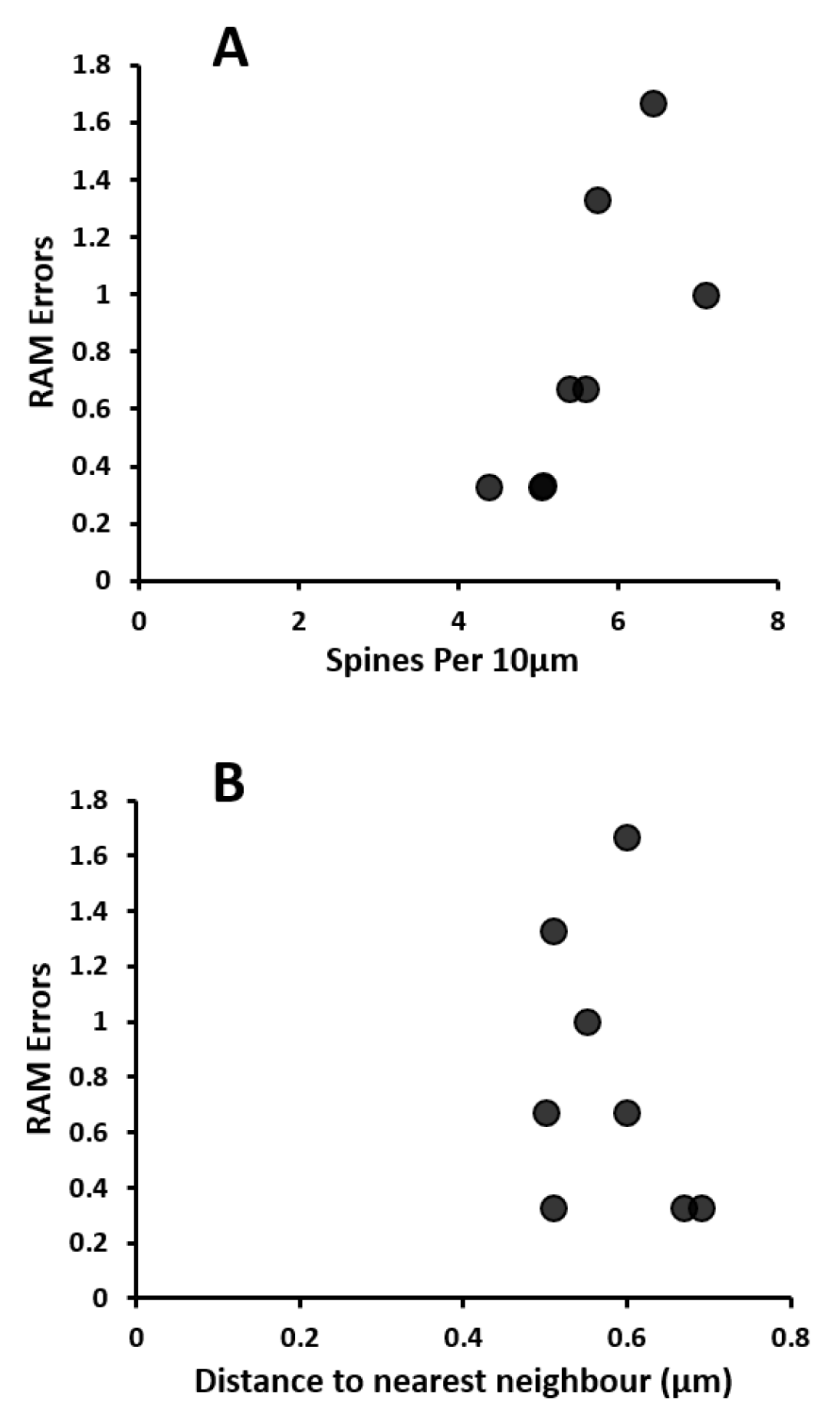
Correlations between spine morphology of CA1 basal dendrites and mean number of errors on the final three trials of the rotated radial-arm maze (RAM) task. ( **A**) Spine density per 10 *µ* of basal dendrites in the CA1 region was not significantly correlated with mean number of errors. ( **B**) Mean distance between spines and their nearest neighbour was not significantly correlated with mean number of errors.

A Bonferroni corrected Pearson’s correlation, using an adjusted-alpha level of 0.025, found no significant correlation between errors on the last three trials of the rotated version of the task and spine density ( *r* = 0.737, *p* = 0.037) or between errors and mean distance between spines ( *r* = -0.315, *p* = 0.448) ( Figure 4).

## Discussion

The present study failed to find an effect of radial-arm maze training on the density or clustering of spines on the basal arbors of CA1 neurons or their clustering. As such, the present results have not replicated findings from a previous study, which showed increased spine density following radial-arm maze training and a correlation between spine density and behavioural performance ^21, 22^. This then raises the question, why the difference across studies?

Hippocampal dendritic spines are temporally dynamic structures and, as such, the time at which they are assessed may be a critical factor in whether or not differences in treatment groups are found. Many behavioural studies do not report the post-training time period that is being assessed, however, from the information that is available, behaviourally induced changes in spines have been found during a range of time periods using single time-point techniques. For example, Mahmmoud *et al.* ^21^ found increased CA1 basal spine density when rats were perfused 6 hours post-training on a radial-arm maze task whilst Harland *et al*. ^19^ reported increased CA1 basal spines when rats that were previously housed in enriched environments were perfused 24h post-training on a radial-arm maze task.

However, Rusakov *et al*. ^3^ found changes in CA1 spine clustering, but no change in density, 6 days after water-maze training. More detailed information on the time-course of CA1 spine formation and turnover can be acquired from slice-studies. Bourne *et al.* ^27^ showed initial plasticity, including spinogenesis along the dendritic shaft of CA1 neurons, following stimulation that was designed to mimic long-term potentiation. However, at 2 hours post-stimulation there was no overall change in spine density suggesting a redistribution of spines and a balance between loss and gain of spines ^27^. A further *in vivo* study showed CA1 spines to be predominantly impermanent with lifespans of approximately 5–15 days ^28^. In our study, we perfused the animals 90 minutes after their last radial-arm maze session. As such, we should be in a position to capture both immediate post-learning spinogenesis as well any longer-lasting changes in numbers or clustering from the previous training sessions.

Another possibility is that the stage of learning is a critical factor in whether behavioural-induced structural changes are observed. The hippocampus appears to be particularly important for initial learning of spatial memory tasks ^22^. It is possible that there was increased spine density and clustering during the early stages of training but this was not maintained for later stages of training. However, other studies have found differences following 10 sessions of training ^21^, which is not dissimilar to the 16 sessions of training used in the present study. Additionally, as spine turnover typically occurs over 5–15 days ^28^, we should also be capturing the effects of earlier training sessions. Furthermore, CA1 activity has been shown to correlate with performance on a radial-arm maze task during late-stage training again suggesting that the stage of learning we assessed was not a critical factor ^17^.

The current study assessed basal CA1 spines but not apical dendrites. This decision was based on previous research where basal CA1 spine density had been shown to increase following spatial memory training (e.g. 19, 29). Data from apical dendrites has yielded mixed results with some studies finding no effect of spatial training (e.g. 19, 29). However Mahmmoud *et al.* (2015) ^21^, found increased CA1 basal and apical spine density when rats were perfused 6h after the last of 10 radial-arm maze sessions. In comparison, Watman and Holahan (2014) ^20^ found increases in basal but not apical spines when animals were sacrificed two days post-training and increased apical, but not basal, spines when they were sacrificed 29 days post-training. These studies raise the possibility that changes in apical spines may reflect long-term changes and, as such, if they had been assessed in the current study they may have provided information about changes that had occurred during the initial stages of training. However, the expectation based on previous studies is that basal spines should still have been sensitive to the protocol employed in the current study. 

It is common practice for studies using optical methods to examine dendritic spines to classify them as thin, mushroom, or stubby according to the spines head and neck diameters ^30^. It has been argued that thin spines are flexible ‘learning spines’ that may change in size or even dismantle rapidly during learning whereas mushroom spines could be more stable ‘memory spines’ ^31^. Therefore, changes in thin and mushroom spines could reflect changes in new learning and acquired mnemonic information, respectively. Indeed, previous studies have reported changes in the proportion and density of CA1 basal thin and mushroom spines following spatial memory training ^19, 21^. However, there is evidence that the typical light microscopy used in the current and previous studies do not have sufficient spatial resolution to properly resolve the distinguishing features of spines. Consistent with this idea, Tønnense *et al.* (2014) ^32^ used super-resolution stimulated emission depletion (STED) imaging and found only a few percent of spines to be stubby. The same data analysed with 2-photon imaging found that spines appearing stubby almost always had short necks when resolved with STED. In the literature, the estimated 20–25% of spines classified as stubby could in fact have short necks that are not readily seen without super-high resolution imaging ^19, 21^. Furthermore, there is a large diversity in the appearance of the spine types. It has been asserted that many cannot be classified due to having intermediate characteristics or characteristics that do not fit within the hypothesized categories ^33^. Therefore, for these reasons the current study did not classify spines into discrete categories as doing this may not be informative. Instead, future studies could measure spine head and neck diameters, length, volume etc using a continuous spectrum as has been suggested elsewhere ^30, 33, 34^.

Another difference across studies is the methodology for assessing spines. Mahmmoud *et al.* ^21^ used DiOlistic labelling in order to stain cells using a fluorescent dye, which may be more sensitive than the Golgi approach used in the present study ^35^. The Golgi method of staining certainly has limitations, as it only stains a small percentage of the total neurons present and there is still uncertainty as to which neurons are stained and why ^36^. As such, the stained neurons may not be representative ^37^ and they may not be sufficiently capturing cells active during the task ^38^. Nevertheless, other studies have used Golgi-stained tissue to show behaviourally-induced changes in spine density in CA1 neurons ^19, 39^ and we have also shown lesion-induced changes in spine number and clustering using the same methodology as that used here ^22^.

## Conclusions

Together, the present results suggest that spatial learning is not always accompanied by changes in either the density or clustering of dendritic spines on the basal arbor of CA1 pyramidal neurons. As such, there is a need for additional research to determine the conditions under which CA1 spinogenesis contributes to spatial learning and memory. Using longitudinal *in vivo* imaging to track the formation and location of new spines across training ^40^ would better enable us to assess how spine dynamics correlate with on-going behavioural performance.

## Data availability

### Underlying data

Figshare: Collection holding data and metadata on CA1 dendritic spine density and clustering in rats following training on a radial-arm maze task, https://doi.org/10.6084/m9.figshare.c.4910244.v1 ^41^.

This project contains the following underlying data:

- Numerical data for radial-arm maze performance, spine density and spine clustering
- Raw and processed images of Golgi-stained dendritic spines

Data are available under the terms of the Creative Commons Zero “No rights reserved” data waiver (CC0 1.0 Public domain dedication).

Access to original slides can be provided upon request to Seralynne Vann (corresponding author; vannsd@cardiff.ac.uk).
