# Supplementary material for: Lack of change in CA1 dendritic spine density or clustering in rats following training on a radial-arm maze task
Source: Wellcome Open Res. 2020 May 15;5:68. Originally published 2020 Apr 14. [Version 2] doi: 10.12688/wellcomeopenres.15745.2 (PMC7236580; doi:10.12688/wellcomeopenres.15745.2)
Supplement: Peer review history

# Peer review: Reviewer response for version 2

**DOI:** 10.21956/wellcomeopenres.17474.r38736

**Published:** 2020-05-18

**Authors:** Michael G. Stewart

**Affiliations:** 1 Department of Life, Health and Chemical Sciences, The Open University, Milton Keynes, UK

This revised manuscript is a very good response to my concerns, meeting each point.

Is the work clearly and accurately presented and does it cite the current literature?

Yes

If applicable, is the statistical analysis and its interpretation appropriate?

Yes

Are all the source data underlying the results available to ensure full reproducibility?

Yes

Is the study design appropriate and is the work technically sound?

Partly

Are the conclusions drawn adequately supported by the results?

Partly

Are sufficient details of methods and analysis provided to allow replication by others?

Yes

Reviewer Expertise:

NA

I confirm that I have read this submission and believe that I have an appropriate level of expertise to confirm that it is of an acceptable scientific standard.

# Peer review: Reviewer response for version 2

**DOI:** 10.21956/wellcomeopenres.17474.r38735

**Published:** 2020-05-18

**Authors:** Bruce Harland

**Affiliations:** 1 School of Pharmacy, University of Auckland, Auckland, New Zealand

I have no further comments.

Is the work clearly and accurately presented and does it cite the current literature?

Yes

If applicable, is the statistical analysis and its interpretation appropriate?

Yes

Are all the source data underlying the results available to ensure full reproducibility?

Yes

Is the study design appropriate and is the work technically sound?

Yes

Are the conclusions drawn adequately supported by the results?

Yes

Are sufficient details of methods and analysis provided to allow replication by others?

Partly

Reviewer Expertise:

Population and single-cell neuronal recording, spatial memory tasks in rodents, neuromorphological analysis, targeted brain lesions and deactivation, treatments for spinal cord injury

I confirm that I have read this submission and believe that I have an appropriate level of expertise to confirm that it is of an acceptable scientific standard.

# Peer review: Reviewer response for version 1

**DOI:** 10.21956/wellcomeopenres.17263.r38425

**Published:** 2020-05-04

**Authors:** Bruce Harland

**Affiliations:** 1 School of Pharmacy, University of Auckland, Auckland, New Zealand

Craig *et al*. investigated whether acquisition of a radial-arm maze task in rats induced increases in CA1 basal dendritic spines. Significant improvement was shown by rats in this behavioral task, and a rotated phase of the experiment provided additional evidence that the task was hippocampally dependent. A yoked control group experienced equivalent locomotion in the maze but without acquisition of the task, providing a robust control alongside home-caged controls. A Golgi stain was used to visualize neuronal structure, a technique that provides very good staining of dendritic spines in particular, which is what was quantified in this study. The technique has some drawbacks, for example, the opaque nature of the stain means that spines above and below the dendrite cannot be visualized. Although dye-labelling techniques overcome this issue, to a degree, they usually have their own drawbacks and limitations. Dorsal CA1 basal dendritic spines were analyzed as a measure of experience-dependent neural plasticity.

 The methods in this paper were well chosen and executed, and suitable for comparison to other studies. Interestingly, Craig *et al*. did not find increases in CA1 basal spine density or clustering reported by some other studies after spatial task acquisition. Of particular note is the fact that there was no difference between the spatially-trained rats and the home-cage controls. They provide a well thought out and comprehensive discussion of why there may be differences between studies. **These findings are of real interest to the field** and highlight the need for further research into the nature of spine dynamics after spatial learning.

 I would like some additional minor clarifications about the methods.

1. The z-distance between images in the image stacks needs to be provided. The example images in Figure 1 suggest that an acceptable ‘step size’ has been used, but this needs to be given.
2. Some mention needs to be made about whether spines above or below the dendrites (in the optical field of the microscope images) were counted or quantified in some way, and if so, how. If not, is usual to state that no attempt was made to count these hidden spines. As the hidden spines are inaccessible in the same way for all of the different groups, it should not affect the quantification.
3. It would be useful to know the orientation of the tissue ‘slices’; coronal, sagittal?
4. Dendritic sections were captured from dorsal CA1, which is a large extent of the hippocampus especially in the rostral-caudal direction. **This is perfectly acceptable**, and is the broad range specified by many other studies. However, the area considered dorsal is not always consistent between studies. **If possible**, can the authors provide any additional information about the range of CA1 used? For example, were dendritic segments captured from where dorsal CA1 first emerges rostrally in coronal sections all the way back to the caudal limits of the dorsal region? This would be potentially useful information for future studies, for instance if it turns out that there are differences in neural plasticity related to spatial learning within dorsal CA1.

Is the work clearly and accurately presented and does it cite the current literature?

Yes

If applicable, is the statistical analysis and its interpretation appropriate?

Yes

Are all the source data underlying the results available to ensure full reproducibility?

Yes

Is the study design appropriate and is the work technically sound?

Yes

Are the conclusions drawn adequately supported by the results?

Yes

Are sufficient details of methods and analysis provided to allow replication by others?

Partly

Reviewer Expertise:

Population and single-cell neuronal recording (including in hippocampus), spatial memory tasks in rodents, neuromorphological analysis, targeted brain lesions and deactivation, treatments for spinal cord injury

I confirm that I have read this submission and believe that I have an appropriate level of expertise to confirm that it is of an acceptable scientific standard.

# Response

**Published:** 2020-05-04

**Authors:** Seralynne Vann

**Affiliations:** Cardiff University, UK

We thank the reviewer for the helpful comments. We have addressed each in turn.

1. **The z-distance between images in the image stacks needs to be provided. The example images in Figure 1 suggest that an acceptable ‘step size’ has been used, but this needs to be given**.
  We have clarified that a 0.2µm step size was used to collect approximately 30 – 90 images per image stack.
2. **Some mention needs to be made about whether spines above or below the dendrites (in the optical field of the microscope images) were counted or quantified in some way, and if so, how. If not, is usual to state that no attempt was made to count these hidden spines. As the hidden spines are inaccessible in the same way for all of the different groups, it should not affect the quantification.**
  We have clarified that spines above or below the dendrite with respect to the field of view are not visible with Golgi stain and hence no attempt to count or correct for them was made.
3. **It would be useful to know the orientation of the tissue ‘slices’; coronal, sagittal?** 
  We have clarified that coronal slices were used.
4. **Dendritic sections were captured from dorsal CA1, which is a large extent of the hippocampus especially in the rostral-caudal direction. This is perfectly acceptable, and is the broad range specified by many other studies. However, the area considered dorsal is not always consistent between studies. If possible, can the authors provide any additional information about the range of CA1 used? For example, were dendritic segments captured from where dorsal CA1 first emerges rostrally in coronal sections all the way back to the caudal limits of the dorsal region? This would be potentially useful information for future** **studies, for instance if it turns out that there are differences in neural plasticity related to spatial learning within dorsal CA1.**
  We have clarified that CA1 basal dendritic segments were imaged from the dorsal region of CA1 extending -2.7mm to -4.6mm posterior to bregma according to the atlas of Paxinos and Watson (2006).

**References**

Paxinos, G., & Watson, C. (2006). *The rat brain in stereotaxic coordinates: hard cover edition*. Elsevier.

# Peer review: Reviewer response for version 1

**DOI:** 10.21956/wellcomeopenres.17263.r38429

**Published:** 2020-04-15

**Authors:** Michael G. Stewart

**Affiliations:** 1 Department of Life, Health and Chemical Sciences, The Open University, Milton Keynes, UK

This is an interesting study which reports that there is a lack of change in CA1 dendritic spine density or clustering in adult rats following training on a radial-arm maze task. The study describes a careful analysis of the behavioural testing and the statistical analyses is provided.

 The measurement of spines has been made from basal dendrites on Golgi stained section of CAI. While the quantitative analyses are reported in detail there are some issues when comparing to previous studies, in particular that of Mahmmoud *et al. *(2015) ^1^.

1. The present study used Golgi staining which can be capricious and examined only basal dendrites in CA1, whereas that of Mahmmoud *et al.* used DiI staining of slices and so ensured that dendrites are more fully impregnated. Moreover, Mahmmoud *et al.* examined both apical and basal dendrites.
2. Dendritic spines can be classified as either thin or mushroom and those without a neck are classified as stubby. Mushroom spines are classified on the basis of head diameter. This method is in agreement with well-accepted methods for spine type classification. However, no attempt was made to classify spine types in this study.
3. Spines can be branched or unbranched, it is not clear whether this was examined.
4. No depth analyses appear to have made for hidden spines which depends on section thickness, and can affect spine counts, it is not clear whether this has been taken into account.
5. Changes after training could affect synapses on spines, increasing spine head diameter, and this might have occurred without spine size changes.

Is the work clearly and accurately presented and does it cite the current literature?

Yes

If applicable, is the statistical analysis and its interpretation appropriate?

Yes

Are all the source data underlying the results available to ensure full reproducibility?

Yes

Is the study design appropriate and is the work technically sound?

Partly

Are the conclusions drawn adequately supported by the results?

Partly

Are sufficient details of methods and analysis provided to allow replication by others?

Yes

Reviewer Expertise:

NA

I confirm that I have read this submission and believe that I have an appropriate level of expertise to confirm that it is of an acceptable scientific standard, however I have significant reservations, as outlined above.

## References

[1] : Spatial and Working Memory Is Linked to Spine Density and Mushroom Spines. *PLoS One*.2015;10(10) : 10.1371/journal.pone.0139739 e0139739 10.1371/journal.pone.0139739 26469788PMC4607435

# Response

**Published:** 2020-04-28

**Authors:** Seralynne Vann

**Affiliations:** Cardiff University, UK

**Comment 1**

**The present study used Golgi staining which can be capricious and examined only basal dendrites in CA1, whereas that of Mahmmoud *et al.* used DiI staining of slices and so ensured that dendrites are more fully impregnated. Moreover, Mahmmoud *et al.* examined both apical and basal dendrites.**

****

We agree that Golgi staining has limitations with regards to the unpredictable nature of staining and had indeed reported this as a limitation of the present study in the Discussion. In our study, dendrites needed to be fully impregnated to be included for analysis. We have now clarified this in the Image Analysis section of the Methods.

Despite the capricious nature of the Golgi method we think this approach is still informative as others have found behaviourally-induced changes in spine density in CA1 (Leuner *et al.*, 2003; Harland *et al.*, 2014) and we have previously shown lesion-induced changes in spine density and clustering in CA1 (Dillingham *et al.*, 2019). This point was also included in the Discussion.

Previous research has indicated that basal CA1 spine density can increase following spatial memory training whereas data from apical dendrites have yielded mixed results. Harland *et al.* (2014) and Moser *et al.* (1997) found basal, but not apical, spines to be sensitive to spatial training. Wartman and Holahan (2014) found increases in basal but not apical spines where animals were sacrificed two days post-training and increased apical, but not basal, spines when they were sacrificed 29 days post-training. This one study raises the possibility that changes in apical spines may reflect long-term changes and, as such, if we had assessed them in our study they may have provided information about changes that had occurred during the initial stages of training. However, the expectation based on previous studies that basal spines should still have been sensitive to our protocol. We have now included this as part of our Discussion.

**Comment 2**

**Dendritic spines can be classified as either thin or mushroom and those without a neck are classified as stubby. Mushroom spines are classified on the basis of head diameter. This method is in agreement with well-accepted methods for spine type classification. However, no attempt was made to classify spine types in this study.**

Previous studies using optical microscopes classified  spines into the categories of thin, mushroom or stubby, first proposed by Peters and Kaiserman‐Abramof (1970). However, there is evidence that the typical light microscopy used in studies such as ours does not have the sufficient spatial resolution to properly resolve the distinguishing features of spines. Tønnesen *et al.* (2014) used super-resolution stimulated emission depletion (STED) imaging and found only a few percent of spines to be stubby. The same data analysed with 2-photon imaging found that spines appearing stubby almost always in fact had short necks when resolved with STED.

In the literature the estimated 20-25% of spines classified as stubby could in fact have short necks that are not readily seen without super-high resolution imaging (Harland *et al.*, 2014; Mahmmoud *et al.*, 2015).  Furthermore, there is a large diversity in the appearance of the spine types. It has been asserted that many cannot be classified due to having intermediate characteristics or characteristics that do not fit within the hypothesised categories (Arellano *et al.*, 2007). Therefore, for these reasons we think classifying spines into discrete categories may not be informative. We have added a paragraph to the Discussion covering this important methodological consideration.

**Comment 3.**

**Spines can be branched or unbranched, it is not clear whether this was examined**.

Where a clearly branched spine was identified, it was counted just once (i.e. branched spines were not double counted). No further examination of these spines was carried out. This is now clarified in the Methods.

**Comment 4**

**No depth analyses appear to have made for hidden spines which depends on section thickness, and can affect spine counts, it is not clear whether this has been taken into account.**

We thanks the Reviewer for highlighting this. We accounted for depth by collecting z-stacks from the bottom through to the top of the dendrite. Our image processing protocol was then able to project these onto a simple plane using the in-built ImageJ temporal colour code hyperstack projection method. The subsequent processed image represents spines at different depths using different colours. This ensures that spines that may otherwise be hidden at different depths are not missed. This is now clearly stated in the Methods section.

**Comment 5**

**Changes after training could affect synapses on spines, increasing spine head diameter, and this might have occurred without spine size changes.**

We thank the Reviewer for raising this point. As mentioned in our response to comment 2, classifying spines into discrete categories is problematic. We think the Reviewer’s suggestions to measure spine head diameter could be appropriate if measured on a continuous spectrum. This could also be extended to included spine neck sizes as has been suggested by others (Yuste & Majewska, 2001; Arellano *et al.*, 2007). We have now included this as a suggestion that future studies could utilise alongside spine density and clustering.

Readers considering this approach should note that over 75% of spines in the hippocampus are small spines with head diameters of <0.6 µm. These spines are more prone to rapid formation and elimination (Bourne & Harris, 2007; Bourne & Harris, 2011; MacDougall & Fine, 2014). This should be taken into consideration when designing experiments for subsequent analysis of hippocampal spine head and neck sizes.

**References**

Arellano, J.I., Benavides-Piccione, R., DeFelipe, J. & Yuste, R. (2007) Ultrastructure of dendritic spines: correlation between synaptic and spine morphologies. *Frontiers in neuroscience*, **1**, 10.

Bourne, J. & Harris, K.M. (2007) Do thin spines learn to be mushroom spines that remember? *Current opinion in neurobiology*, **17**, 381-386.

Bourne, J.N. & Harris, K.M. (2011) Coordination of size and number of excitatory and inhibitory synapses results in a balanced structural plasticity along mature hippocampal CA1 dendrites during LTP. *Hippocampus*, **21**, 354-373.

Dillingham, C.M., Milczarek, M.M., Perry, J.C., Frost, B.E., Parker, G.D., Assaf, Y., Sengpiel, F., O'Mara, S.M. & Vann, S.D. (2019) Mammillothalamic Disconnection Alters Hippocampocortical Oscillatory Activity and Microstructure: Implications for Diencephalic Amnesia. *The Journal of Neuroscience*, **39**, 6696-6713.

Harland, B.C., Collings, D.A., McNaughton, N., Abraham, W.C. & Dalrymple-Alford, J.C. (2014) Anterior thalamic lesions reduce spine density in both hippocampal CA1 and retrosplenial cortex, but enrichment rescues CA1 spines only. *Hippocampus*, **24**, 1232-1247.

Leuner, B., Falduto, J. & Shors, T.J. (2003) Associative memory formation increases the observation of dendritic spines in the hippocampus. *Journal of Neuroscience*, **23**, 659-665.

MacDougall, M.J. & Fine, A. (2014) The expression of long-term potentiation: reconciling the preists and the postivists. *Philosophical Transactions of the Royal Society B: Biological Sciences*, **369**, 20130135.

Mahmmoud, R.R., Sase, S., Aher, Y.D., Sase, A., Groger, M., Mokhtar, M., Hoger, H. & Lubec, G. (2015) Spatial and Working Memory Is Linked to Spine Density and Mushroom Spines. *PLoS One*, **10**, e0139739.

Moser, M.B., Trommald, M., Egeland, T. & Andersen, P. (1997) Spatial training in a complex environment and isolation alter the spine distribution differently in rat CA1 pyramidal cells. *Journal of Comparative Neurology*, **380**, 373-381.

Peters, A. & Kaiserman‐Abramof, I.R. (1970) The small pyramidal neuron of the rat cerebral cortex. The perikaryon, dendrites and spines. *American Journal of Anatomy*, **127**, 321-355.

Tønnesen, J., Katona, G., Rózsa, B. & Nägerl, U.V. (2014) Spine neck plasticity regulates compartmentalization of synapses. *Nature Neuroscience*, **17**, 678-685.

Wartman, B.C. & Holahan, M.R. (2014) The impact of multiple memory formation on dendritic complexity in the hippocampus and anterior cingulate cortex assessed at recent and remote time points. *Frontiers in Behavioral Neuroscience*, **8**, 128.

Yuste, R. & Majewska, A. (2001) Book Review: On the Function of Dendritic Spines. *The Neuroscientist*, **7**, 387-395.
